# *Mycoplasma genitalium* prevalence, antimicrobial resistance-associated mutations, and coinfections with non-viral sexually transmitted infections in high-risk populations in Guatemala, Malta, Morocco, Peru and South Africa, 2019–2021

**DOI:** 10.3389/fmicb.2023.1130762

**Published:** 2023-02-22

**Authors:** Elena Shipitsyna, Ranmini Kularatne, Daniel Golparian, Etienne E. Müller, Silver K. Vargas, Ronza Hadad, Valeska Padovese, Amina Hancali, Christian S. Alvarez, Hicham Oumzil, Elsy Camey, Karel Blondeel, Igor Toskin, Magnus Unemo

**Affiliations:** ^1^World Health Organization Collaborating Centre for Gonorrhoea and Other STIs, Department of Laboratory Medicine, Microbiology, Faculty of Medicine and Health, Örebro University, Örebro, Sweden; ^2^Department of Medical Microbiology, D.O. Ott Research Institute of Obstetrics, Gynecology and Reproductology, St. Petersburg, Russia; ^3^Labtests Laboratory and Head Office, Mt Wellington, Auckland, New Zealand; ^4^Department of Clinical Microbiology & Infectious Diseases, Faculty of Health Sciences, University of the Witwatersrand, Johannesburg, South Africa; ^5^Centre for HIV and STIs, National Institute for Communicable Diseases, National Health Laboratory Service, Johannesburg, South Africa; ^6^School of Public Health and Administration, Centre for Interdisciplinary Investigation in Sexuality, AIDS, Society and Laboratory of Sexual Health, Universidad Peruana Cayetano Heredia, Universidad Peruana Cayetano Heredia, Lima, Peru; ^7^Genitourinary Clinic, Department of Dermatology and Venereology, Mater Dei Hospital, Msida, Malta; ^8^STIs Laboratory, National Institute of Hygiene, Ministry of Health, Rabat, Morocco; ^9^Sida y Sociedad ONG (SISO), Escuintla, Guatemala; ^10^Faculty of Medicine and Pharmacy, University Mohamed V, Rabat, Morocco; ^11^Department of Sexual and Reproductive Health and Research, World Health Organization, Geneva, Switzerland; ^12^Faculty of Medicine and Health Sciences, Ghent University, Ghent, Belgium; ^13^Institute for Global Health, University College London (UCL), London, United Kingdom

**Keywords:** *M. genitalium* prevalence and antimicrobial resistance, Mycoplasma genitalium, men who have sex with men, women at-risk, prevalence, antimicrobial resistance, azithromycin, moxifloxacin

## Abstract

The prevalence of *Mycoplasma genitalium* (MG) and MG antimicrobial resistance (AMR) appear to be high internationally, however, prevalence data remain lacking globally. We evaluated the prevalence of MG and MG AMR-associated mutations in men who have sex with men (MSM) in Malta and Peru and women at-risk for sexually transmitted infections in Guatemala, South Africa, and Morocco; five countries in four WHO regions mostly lacking MG prevalence and AMR data, and estimated MG coinfections with *Chlamydia trachomatis* (CT), *Neisseria gonorrhoeae* (NG), and *Trichomonas vaginalis* (TV). Male urine and anorectal samples, and vaginal samples were tested for MG, CT, NG, and TV (only vaginal samples) using Aptima assays (Hologic). AMR-associated mutations in the MG 23S rRNA gene and *parC* gene were identified using ResistancePlus MG kit (SpeeDx) or Sanger sequencing. In total, 1,425 MSM and 1,398 women at-risk were recruited. MG was detected in 14.7% of MSM (10.0% in Malta and 20.0% Peru) and in 19.1% of women at-risk (12.4% in Guatemala, 16.0% Morocco, 22.1% South Africa). The prevalence of 23S rRNA and *parC* mutations among MSM was 68.1 and 29.0% (Malta), and 65.9 and 5.6% (Peru), respectively. Among women at-risk, 23S rRNA and *parC* mutations were revealed in 4.8 and 0% (Guatemala), 11.6 and 6.7% (Morocco), and 2.4 and 3.7% (South Africa), respectively. CT was the most frequent single coinfection with MG (in 2.6% of MSM and 4.5% of women at-risk), compared to NG + MG found in 1.3 and 1.0%, respectively, and TV + MG detected in 2.8% of women at-risk. In conclusion, MG is prevalent worldwide and enhanced aetiological MG diagnosis, linked to clinical routine detection of 23S rRNA mutations, in symptomatic patients should be implemented, where feasible. Surveillance of MG AMR and treatment outcome would be exceedingly valuable, nationally and internationally. High levels of AMR in MSM support avoiding screening for and treatment of MG in asymptomatic MSM and general population. Ultimately, novel therapeutic antimicrobials and/or strategies, such as resistance-guided sequential therapy, and ideally an effective MG vaccine are essential.

## Introduction

*Mycoplasma genitalium* (MG) is a frequent cause of non-gonococcal urethritis in men, and is associated with urethritis/cervicitis, pelvic inflammatory disease, endometritis and likely infertility in women (Lis et al., 2015; Horner and Martin, 2017). MG prevalence data are mainly available in North America (United States and Canada), European Union (mostly Scandinavia and the United Kingdom), Australia, and Japan (Baumann et al., 2018). Accordingly, MG prevalence and antimicrobial resistance (AMR) data are mainly absent in the World Health Organisation (WHO) African Region, WHO Eastern Mediterranean Region, and Latin America and the Caribbean belonging to the WHO Region of Americas (Baumann et al., 2018; Machalek et al., 2020; Jensen et al., 2022). Additionally, in many countries worldwide aetiological diagnosis of MG infections is lacking, and mainly syndromic management is used (Wi et al., 2019).

Similar to other international or national MG management guidelines, the 2021 European guideline on the management of MG infections recommends, for diagnosed MG infections, macrolides (azithromycin/josamycin) as first-line treatment in the absence of macrolide resistance-associated mutations (MRAMs) and the fluoroquinolone moxifloxacin when MRAMs are detected or as second-line treatment for cases with macrolide treatment failures (Jensen et al., 2022). The resistance in MG to macrolides has been rising dramatically during the last decade internationally (Machalek et al., 2020). Also, the resistance to fluoroquinolones has increased in several settings, and occasional untreatable cases are already being identified (Machalek et al., 2020). MG resistance to macrolides is predominantly caused by 23S rRNA gene mutations at nucleotide position A2058 or A2059 (*Escherichia coli* numbering), and moxifloxacin resistance is primarily mediated by mutations in ParC S83 and D87 codons (MG numbering) (Unemo and Jensen, 2017). Appropriate surveillance data on AMR-associated mutations in MG are essential for effective management and control of MG infections, but global AMR data remain very scarce (Machalek et al., 2020). However, a Global MG AMR programme, in line with the WHO Global Gonococcal Antimicrobial Surveillance Programme (WHO GASP) (Unemo et al., 2021), has been discussed.

The Aptima MG assay (Hologic, San Diego, United States) was the first MG diagnostic assay that obtained US FDA approval and this NAAT has been shown to have 20–25% higher sensitivity, with retention of high specificity, compared to previously used diagnostic PCRs (Unemo et al., 2018; Shipitsyna and Unemo, 2020). Comprehensive global prevalence data on MG infections and MG resistance to macrolides and fluoroquinolones are essential for public health purposes, to guide evidence-based prevention, management and control of MG infections, and to inform refinements of aetiologically-based management guidelines as well as syndromic management guidelines.

The present study investigated the prevalence of MG infections in sexually transmitted infection (STI) clinic attendees [men who have sex with men (MSM) and women at-risk] using the Aptima MG assay (Hologic) in five countries belonging to four different WHO Regions Malta (WHO European Region), Guatemala and Peru (Latin America belonging to WHO Region of the Americas), South Africa (WHO African Region), and Morocco (WHO Eastern Mediterranean Region), which are all with exception of South Africa (Muller et al., 2019; Mahlangu et al., 2022; Kularatne et al., 2022a,b) completely lacking MG prevalence and AMR data. Furthermore, the prevalences of MRAMs in the MG 23S rRNA gene and quinolone resistance-associated mutations (QRAMs) in the MG *parC* gene (ParC S83 and D87 codons) were evaluated, and coinfections of MG with *Chlamydia trachomatis* (CT), *Neisseria gonorrhoeae* (NG), and *Trichomonas vaginalis* (TV) were assessed.

## Materials and methods

### Study population and specimens

This is an ancillary study to the comprehensive global clinical evaluation of point-of-care tests (POCTs), for non-viral sexually transmitted infections (STIs) conducted within the WHO initiative global ProSPeRo (global Project on STI POCT) Network in 14 countries across 27 data collection sites^1^ and, accordingly, study participants were recruited through this ProSPeRo study for STI testing. Briefly, consecutive participants were recruited from October 2018 to January 2022 when attending for standard care and/or STI screening at six STI settings in five countries: Genitourinary Clinic, Mater Dei Hospital, Msida, Malta (included asymptomatic and symptomatic MSM); Centro de Salud Alberto Barton and Tahuantinsuyo Bajo, Lima, Peru (asymptomatic and symptomatic MSM); Obstetric and Gynaecological Clinic of the Regional National Hospital of Escuintla, Escuintla, Guatemala [asymptomatic and symptomatic (vast majority) women at-risk]; Centre d’Information et de Dépistage Anonyme et Gratuit, Association Marocaine de Lutte contre le Sida, Rabat, Morocco [asymptomatic (majority) and symptomatic female commercial sex workers]; and Site C Youth Clinic, Khayelitsha district, Cape Town, South Africa [asymptomatic (vast majority) and symptomatic sexually-active adolescent girls and young women aged 18–25 years]. Detailed study participant metadata (demographic and clinical) will be presented in the main WHO ProSPeRo study.^1^ Clinical specimens collected included urine in Aptima Urine Specimen Transport Tube (Hologic), self-collected anorectal swabs from MSM in Aptima Multitest Swab Specimen Collection Kit (Peru and Malta) and vaginal swabs (clinician/self-collected) from women at-risk in Aptima Vaginal Swab Specimen Collection tube (Hologic; Guatemala, Morocco, and South Africa). Specimens from Guatemala, Malta, Morocco, and Peru were stored at -70-80°C prior to shipment to the WHO Collaborating Centre for Gonorrhoea and Other STIs (WHO CC), Sweden for testing, while specimens from South Africa were tested at the STI Section, Centre for HIV and STIs, National Institute for Communicable Diseases (NICD), Johannesburg, South Africa.

### Testing for MG, CT, NG, TV, and AMR-associated mutations in MG

Testing of male urine and anorectal swabs for MG and CT/NG, and vaginal swabs for MG, CT/NG, and TV was performed using Aptima MG, Aptima Combo 2, and Aptima TV assays (Hologic), respectively, on the Panther Platform (Hologic), in accordance to the manufacturer’s instructions (Hologic).

Specimens from Guatemala, Malta, Morocco, and Peru were examined for AMR-associated mutations at the WHO CC, Sweden, while South African specimens were examined at the NICD, South Africa. Genomic DNA was extracted from all MG-positive Aptima specimens using the QIASymphony DSP virus/pathogen kit (Qiagen GmbH, Hilden, Germany) on the QIASymphony instrument (Qiagen) at the WHO CC, Sweden and using an automated DNA extractor, QIAcube HT (Qiagen) at the NICD, South Africa, in accordance to the instructions of the manufacturer (Qiagen). At the WHO CC, Sweden, MRAMs were identified using the ResistancePlus MG kit (SpeeDx Pty. Ltd., NSW, Australia; Tabrizi et al., 2017; Murray et al., 2020a,b; Le Roy et al., 2020) and QRAMs detected with PCR amplification of a 220 bp region of MG *parC* followed by Sanger sequencing, using previously described method (Shipitsyna et al., 2017; Hadad et al., 2018; Unemo et al., 2018; Hilmarsdóttir et al., 2020) and PCR primers (Deguchi et al., 2001; Shimada et al., 2010). At the NICD, South Africa, MRAMs were identified using PCR amplification of a 147 bp region of the MG 23S rRNA gene followed by Sanger sequencing using previously described primers (Jensen et al., 2008), and methods (Muller et al., 2019; Mahlangu et al., 2022). QRAMs were detected by PCR amplification of a 220 bp region of MG *parC* followed by Sanger sequencing using previously described primers (Deguchi et al., 2001; Shimada et al., 2010), as previously described (Muller et al., 2019; Mahlangu et al., 2022).

### Statistics

Prevalence of MG and MG AMR-associated mutations and coinfections with CT, NG, and TV were computed with 95% confidence intervals (CI 95%) for each setting and sample type. In MSM, a composite MG patient status was determined, i.e., a positive MG infection patient status was defined as MG detected in ≥1 of the anatomical sites screened. Differences in prevalence of MG and MG AMR-associated mutations were estimated using chi-square statistics, or, when required, Fisher’s exact test. All calculations were done with the StatPlus (AnalystSoft) software. Significance was set at *p* < 0.05.

### Ethics approval

The study was approved by the corresponding Ethics Boards at the participating countries/settings, with written and verbal informed consent obtained from all study participants. All tested samples were anonymised and no personal identification data were available in the study.

## Results

### Prevalence of MG

A total of 2,823 study participants (1,425 MSM and 1,398 women at-risk) were recruited. Of the 1,425 males, 659 were enrolled in Peru and 766 in Malta. Of the 1,398 females, 178 were recruited in Guatemala, 409 in Morocco, and 811 in South Africa.

For MG prevalence testing, 16 rectal, 39 urine, 22 vaginal samples were not available for testing, and 12 rectal, two urine, 17 vaginal samples were invalid (reported as invalid by the Panther Platform or lacking sufficient volume for the Aptima MG assay) for evaluation. All these specimens (*n* = 108) were excluded from the analysis. Of the remaining 1,397 rectal samples and 1,384 urine samples from MSM, 173 (12.4%) and 60 (4.3%), respectively, were MG positive (Table 1). MG was significantly more prevalent among MSM in Peru (composite MG patient status 20.0%) as compared to Malta (10.0%; *p* < 0.0001). Furthermore, MG was significantly more frequent in anorectal samples (12.4%) than in urine samples (4.3%) (*p* < 0.0001). Thus, 71.0% (147 of 207) of MG-infected MSM were only detected by testing anorectal swabs and had MG-negative urine samples (Table 1).

**Table 1:** Prevalence of *Mycoplasma genitalium* in high-risk populations in five countries in four WHO Regions.

Population/ specimen	% [CI 95%] (No. of positive tests/No. of valid tests)					
	Malta	Peru	Guatemala	Morocco	South Africa	Total
MSM						
Anorectal samples	8.1 [6.3–10.3] (60/744)	17.3 [14.5–20.5] (113/653)				12.4 [10.7–14.3] (173/1397)
Urine samples	3.3 [2.2–5.0] (24/726)	5.5 [3.9–7.6] (36/658)				4.3 [3.4–5.6] (60/1384)
MG patient status	10.0 [8.0–12.5] (75/747)	20.0 [17.1–23.3] (132/659)				14.7 [12.9–16.7] (207/1406)
Women at-risk						
Vaginal samples			12.4 [8.1–18.3] (22/178)	16.0 [12.6–20.1] (63/393)	22.1 [19.3–25.2] (174/788)	19.1 [17.0–21.3] (259/1359)

WHO, World Health Organisation; CI, confidence interval; No., number; MSM, men who have sex with men.

In women at-risk, 259 of the 1,359 (19.1%) vaginal samples were MG positive. MG was significantly more prevalent in South Africa (22.1%) than in Guatemala (12.4%; *p* = 0.0149) and Morocco (16.0%; *p* = 0.0440), with no significant difference between Guatemala and Morocco.

### Prevalence of MG AMR-associated mutations

Of the 173 rectal, 60 urine, and 259 vaginal samples positive for MG, 107 (61.8%), 35 (58.3%), and 105 (40.5%) samples, respectively, were successfully tested for MRAMs, and 101 (58.4%), 17 (28.3%), and 98 (37.8%) samples, respectively, for QRAMs (Table 2). In MSM in Malta and Peru, the overall prevalence (composite MG patient status) of MRAMs was very high (66.7%), with no significant difference between the countries (68.1% vs. 65.9%; Table 2). QRAMs were significantly more prevalent in Malta as compared to Peru (29.0% vs. 5.6%, *p* = 0.0104). Dual resistance mutations were also more frequent in Malta compared to in Peru (20.7% vs. 4.6%, *p =* 0.0707), but the difference did not reach statistical significance. In women at-risk in Guatemala, Morocco and South Africa, the prevalence of MRAMs, QRAMs, and dual resistance mutations were 6.7% (range, 2.4–11.6%), 4.1% (range, 0–6.7%), and 1.5% (range, 0–3.3%), respectively (Table 2), with no significant differences (likely because of the low number of samples) between the countries.

**Table 2:** Prevalence of *M. genitalium* macrolide and quinolone resistance-associated mutations in high-risk populations in five countries in four WHO Regions.

	% [CI 95%] (No. of positive tests/No. of valid tests)						Malta	Peru	Guatemala	Morocco	South Africa	Total
MSM						
Anorectal samples						
Typeable for 23S rRNA mutations	60.0% (36/60)	62.8% (71/113)				61.8% (107/173)
23S rRNA mutations^a^	72.2 [54.6–85.2] (26/36)	63.4 [51.1–74.3] (45/71)				66.4 [56.5–75.0] (71/107)
Typeable for *parC* mutations	48.3% (29/60)	63.7% (72/113)				58.4% (101/173)
ParC S83 or D87 mutations	24.1 [11.0–44.0] (7/29)	4.2 [1.1–12.5] (3/72)				9.9 [5.1–17.9] (10/101)
ParC S83I	13.8 [4.5–32.6] (4/29)	0 [0–6.3] (0/72)				4.0 [1.3–10.4] (4/101)
ParC S83N	6.9 [1.2–24.2] (2/29)	1.4 [0.1–8.5] (1/72)				3.0 [0.8–9.1] (3/101)
ParC D87N	3.5 [0.2–19.6] (1/29)	2.8 [0.5–10.6] (2/72)				3.0 [0.8–9.1] (3/101)
Dual resistance mutations^b^	17.9 [6.8–37.6] (5/28)	1.5 [0.1–9.3] (1/66)				6.4 [2.6–13.9] (6/94)
Urine samples						
Typeable for 23S rRNA mutations	62.5% (15/24)	55.6% (20/36)				58.3% (35/60)
23S rRNA mutations^a^	53.3 [27.4–77.7] (8/15)	45.0 [23.8–68.0] (9/20)				48.6 [31.7–65.7] (17/35)
Typeable for *parC* mutations	20.8% (5/24)	33.3% (12/36)				28.3% (17/60)
ParC S83 or D87 mutations	40.0 [7.3–83.0] (2/5)	8.3 [0.4–40.2] (1/12)				17.7 [4.7–44.2] (3/17)
ParC S83I	20.0 [1.1–70.1] (1/5)	0 [0–30.1] (0/12)				5.9 [0.3–30.8] (1/17)
ParC D87N	20.0 [1.1–70.1] (1/5)	0 [0–30.1] (0/12)				5.9 [0.3–30.8] (1/17)
ParC D87H	0 [0–53.7] (0/5)	8.3 [0.4–40.2] (1/12)				5.9 [0.3–30.8] (1/17)
Dual resistance mutations^b^	20.0 [1.1–70.1] (1/5)	9.1 [0.5–42.9] (1/11)				12.5 [2.2–39.6] (2/16)
MG patient status						
23S rRNA mutations^a^	68.1 [52.8–80.5] (32/47)	65.9 [54.5–75.7] (54/82)				66.7 [57.8–74.6] (86/129)
ParC S83 or D87 mutations	29.0 [14.9–48.2] (9/31)	5. 6 [1.8–14.4] (4/72)				12.6 [7.2–21.0] (13/103)
Dual resistance mutations^b^	20.7 [8.7–40.3] (6/29)	4.6 [0.8–16.7] (2/44)				11.0 [5.2–21.0] (8/73)
Women at-risk						
Vaginal samples						
Typeable for 23S rRNA mutations			95.5% (21/22)	68.3% (43/63)	23.6% (41/174)	40.5% (105/259)
23S rRNA mutations^a^			4.8 [0.3–25.9] (1/21)	11.6 [4.4–25.9] (5/43)	2.4 [0.1–14.4] (1/41)	6.7 [3.0–13.7] (7/105)
Typeable for *parC* mutations			63.6% (14/22)	47.6% (30/63)	31.0 (54/174)	37.8% (98/259)
ParC S83 or D87 mutations			0 [0–26.8] (0/14)	6.7 [1.2–23.5] (2/30)	3.7 [0.6–13.8] (2/54)	4.1 [1.3–10.7] (4/98)
ParC S83I			0 [0–26.8] (0/14)	0 [0–14.1] (0/30)	3.7 [0.6–13.8] (2/54)	2.0 [0.4–7.9] (2/98)
ParC D87N			0 [0–26.8] (0/14)	6.7 [1.2–23.5] (2/30)	0 [0–8.3] (0/54)	2.0 [0.4–7.9] (2/98)
Dual resistance mutations^b^			0 [0–26.8] (0/14)	3.3 [0.2–19.1] (1/30)	0 [0–17.8] (0/23)	1.5 [0.1–9.1] (1/67)

WHO, World Health Organisation; CI, confidence interval; No., number; MSM, men who have sex with men.

a 23S rRNA A2058G, A2059G, A2058T, and A2058C gene mutations are detected combined using the ResistancePlus MG kit (SpeeDx Pty. Ltd, NSW, Australia; Le Roy et al., 2020).

b Mutations in both 23S rRNA A2058/A2059 and ParC S83/D87.

### Prevalence of CT, NG, TV and coinfections with MG

The prevalence of CT and NG in MSM was 14.4% (197/1370, range, 14.1–14.7%) and 8.8% (121/1372, range, 7.3–10.5%), respectively. In women at-risk, CT, NG, and TV were detected in 29.5% (405/1371; range, 13.5–37.9%), 12.6% (172/1365; range, 1.1–20.2%), and 22.2% (303/1368; range, 10.7–39.8%) of women, respectively. Table 3 summarises the prevalence of MG coinfection with CT, NG, and TV for the study participants with known status for all STIs tested (1,364 MSM and 1,348 women at-risk). MG/CT and MG/NG coinfections were detected in 2.6% (range, 2.1–3.2%) and 1.3% (range, 0.8–1.7%) of MSM, respectively. In women at-risk, MG/CT, MG/NG, and MG/TV coinfections were found in 4.5% (range, 1.3–6.8%), 1.0% (range, 0–1.7%), and 2.8% (range, 1.9–4.9%) of women, respectively. Combinations of MG/CT/NG, MG/CT/TV, and MG/NG/TV were revealed in 1.5% (range, 0–2.2%), 2.1% (1.1–3.6%), and 0.1% (range, 0–0.6%) of women at-risk, respectively. In five (0.4%) women (one from Morocco and four from South Africa), all four STI pathogens were detected (Table 3).

**Table 3:** Infections with *M. genitalium*, *Chlamydia trachomatis, Neisseria gonorrhoeae,* and *Trichomonas vaginalis*, including coinfections, in high-risk populations in five countries in four WHO Regions.

STI agents	% [CI 95%], No. of study participants						
	MSM			Women at-risk			
	Malta (*n*=714^a^)	Peru (*n*=650^a^)	All MSM (*n*=1364^a^)	Guatemala (*n*=178^a^)	Morocco (*n*=392^a^)	South Africa (*n*=778^a^)	All women at-risk (*n*=1348^a^)
MG only	7.1 [5.4–9.3], 51	14.2 [11.6–17.1], 92	10.5 [8.9–12.3], 143	6.8 [3.7–11.8], 12	5.4 [3.4–8.2], 21	7.7 [6.0–9.9], 60	6.9 [5.6–8.4], 93
MG and CT	2.1 [1.2–3.5], 15	3.2 [2.1–5.0], 21	2.6 [1.9–3.7], 36	1.7 [0.4–5.3], 3	1.3 [0.5–3.1], 5	6.8 [5.2–8.9], 53	4.5 [3.5–5.8], 61
MG and NG	0.8 [0.3–1.9], 6	1.7 [0.9–3.1], 11	1.3 [0.8–2.0], 17	0 [0–2.6], 0	0 [0–1.2], 0	1.7 [0.9–2.9], 13	1.0 [0.5–1.7], 13
MG and TV	NA	NA	NA	2.3 [0.7–6.0], 4	4.9 [3.0–7.6], 19	1.9 [1.1–3.2], 15	2.8 [2.0–3.9], 38
MG, CT, and NG	0 [0–0.7], 0	0.8 [0.3–1.9], 5	0.4 [0.1–0.9], 5	0 [0–2.6], 0	0.8 [0.2–2.4], 3	2.2 [1.3–3.6], 17	1.5 [0.9–2.3], 20
MG, CT, and TV	NA	NA	NA	1.1 [0.2–4.4], 2	3.6 [2.0–6.1], 14	1.5 [0.8–2.8], 12	2.1 [1.4–3.0], 28
MG, NG, and TV	NA	NA	NA	0.6 [0.03–3.6], 1	0 [0–1.2], 0	0 [0–0.6], 0	0.1 [0–0.5], 1
MG, CT, NG, and TV	NA	NA	NA	0 [0–2.6], 0	0.3 [0.01–1.7], 1	0.5 [0.2–1.4], 4	0.4 [0.1–0.9], 5
CT only	9.2 [7.3–11.7], 66	7.7 [5.8–10.1], 50	8.5 [7.1–10.1], 116	7.9 [4.5–13.1], 14	7.1 [4.9–10.3], 28	15.7 [13.2–18.5], 122	12.2 [10.5–14.1], 164
CT and NG	2.5 [1.6–4.0], 18	2.9 [1.8–4.6], 19	2.7 [1.9–3.8], 37	0.6 [0.03–3.6], 1	0.3 [0.01–1.7], 1	7.2 [5.5–9.3], 56	4.3 [3.3–5.6], 58
CT and TV	NA	NA	NA	2.3 [0.7–6.1], 4	6.6 [4.5–9.7], 26	2.7 [1.7–4.2], 21	3.8 [2.9–5.0], 51
CT, NG, and TV	NA	NA	NA	0 [0–2.6], 0	0.5 [0.1–2.0], 2	1.5 [0.8–2.8], 12	1.0 [0.6–1.8], 14
NG only	3.6 [2.4–5.4], 26	5.1 [3.6–7.1], 33	4.3 [3.3–5.6], 59	0 [0–2.6], 0	1.0 [0.3–2.8], 4	5.5 [4.1–7.4], 43	3.5 [2.6–4.7], 47
NG and TV	NA	NA	NA	0 [0–2.6], 0	0 [0–1.2], 0	1.4 [0.7–2.6], 11	0.8 [0.4–1.5], 11
TV only	NA	NA	NA	4.5 [2.1–9.0], 8	24.2 [20.1–28.8], 95	6.2 [4.6–8.2], 48	11.2 [9.6–13.0], 151

WHO, World Health Organization; CI, confidence interval; No., number; MSM, men who have sex with men; STI, sexually transmitted infection; MG, Mycoplasma genitalium; CT, Chlamydia trachomatis; NG, Neisseria gonorrhoeae; TV, Trichomonas vaginalis; NA, not applicable.

a No. of study participants with known status for MG, CT, and NG (MSM) and for MG, CT, NG, and TV (women at-risk).

## Discussion

The present study including five countries in four WHO regions (on three continents), mostly lacking MG prevalence and AMR data, revealed a high MG prevalence, with significant geographical variations, in MSM (14.7%) and women at-risk (19.1%). In MSM, MG positivity was significantly higher in anorectal samples than in urine samples. The prevalence of MRAMs among MSM was very high in both Malta (68.1%) and Peru (65.9%), whereas QRAMs were significantly less frequent in Peru (5.6%) as compared to Malta (29.0%). Dual-resistance mutations were also much less prevalent in Peru compared to in Malta (4.6% vs. 20.7%), although this difference did not reach significance due to the low number of samples. In women at-risk, the prevalence of MRAMs, QRAMs, and dual resistance mutations were substantially lower (6.7, 4.1, and 1.5%, respectively), with no significant geographical variation. High and considerably varying prevalence of also the other non-viral STIs were recorded in both MSM and women at-risk.

The MG prevalence in asymptomatic and symptomatic MSM attending STI clinics in this study (20.0% in Peru and 10.0% in Malta) were substantially higher than those estimated in community-based (3.2%) and clinic-based (3.7%) studies in a recent meta-analysis (Baumann et al., 2018). However, most recent studies reveal higher MG prevalence estimates among MSM; e.g., ranging from 6.9 to 19.0% in Belgium (Van Praet et al., 2020), France (Berçot et al., 2021), Australia (Couldwell et al., 2018; Read et al., 2019), and Germany (Jansen et al., 2020; Streeck et al., 2022). The considerable variance in the MG prevalence in different studies can be explained by geographical variance but also methodological differences. For example, in our study and the German studies (Jansen et al., 2020; Streeck et al., 2022), reporting the highest MG prevalences, the Aptima MG assay, which has been shown to have 20–25% higher sensitivity compared to diagnostic MG PCRs (Unemo et al., 2018; Shipitsyna and Unemo, 2020; Salado-Rasmussen et al., 2022), was utilized.

In this study, MG positivity in MSM in anorectal samples (12.4%) was substantially higher than in urine samples (4.3%), and 71% of MG-infected MSM would have been missed if anorectal swabs had not been tested. The substantially higher MG prevalence in anorectal compared to urine samples in MSM is in line with studies from Australia (Couldwell et al., 2018; Read et al., 2019) and Germany (Jansen et al., 2020; Streeck et al., 2022). However, in a recent French study the prevalence of MG in MSM was higher in urine (6.3%) as compared to anorectal samples (4.3%; Berçot et al., 2021). Notably, in this study (Berçot et al., 2021) different MG diagnostic methods were used for the divergent sample types, i.e., the *C. trachomatis/Ureaplasma/M. genitalium* Real-TM kit (Sacace, Biotechnologies, Italy) and Cobas TV/MG kit (Roche Diagnostics, United States) for urine and anorectal samples, respectively.

Among women at-risk, a high MG prevalence was revealed in all participating countries. The prevalence of MG in Guatemala (12.4%) and South Africa (22.1%) were mainly in line with the very few previous reports from Latin America and sub-Saharan Africa (Baumann et al., 2018; Jarolimova et al., 2022; Kularatne et al., 2022b). The present study is the first to estimate MG prevalence in a high-risk female population (female commercial sex workers) in the WHO Eastern-Mediterranean region, i.e., in Morocco, which was shown to be high (13.5%).

A recent systematic review and meta-analysis regarding the prevalence of MRAMs and QRAMs in MG showed that global macrolide resistance increased significantly, from 10.0% before 2010 to 51.4% in 2016–2017, whereas fluoroquinolone resistance prevalence remained relatively stable at around 8% (Machalek et al., 2020). Furthermore, substantial geographical variations in the prevalence of MRAMs and QRAMs were observed, with the highest MRAM prevalence in the WHO Western Pacific (68%) and Americas (67%) regions, and highest QRAM prevalence in Western Pacific region (14.3%; Machalek et al., 2020). In Europe, according to a recent review (Fernández-Huerta et al., 2020), many countries have MRAM prevalence >50%, whereas QRAM prevalence is around 5%. However, this review included few studies of MSM. MSM appear significantly more likely to harbour macrolide-resistant MG infections than other populations (Couldwell et al., 2018; McIver et al., 2019; Bradley et al., 2020; Berçot et al., 2021; De Salazar et al., 2021; De Baetselier et al., 2022). In Malta, which has been lacking any MG AMR data hitherto, MRAMs, QRAMs and dual resistance in MSM were detected at prevalence of 68.1, 29.0 and 20.7%, respectively. Similar high levels of MRAMs, QRAMs and dual resistance were recently reported among MSM in Spain (De Salazar et al., 2021), Germany (Dumke et al., 2019), and Belgium (De Baetselier et al., 2021). Extraordinary high prevalence of MRAMs and QRAMs, i.e., 95.2 and 35.7%, respectively, have been found in MSM with recurrent STIs in a recent Belgian study (De Baetselier et al., 2022). MSM, especially those recurrently infected with STIs, are assumed to play a key role in the emergence of AMR in MG (De Baetselier et al., 2022), therefore the use of macrolides and fluoroquinolones should preferably be restricted in this population so as to prevent further emergence of dual-resistant MG infections. However, in the present study it is important to note that 6.9 and 1.4% of the samples in Malta and Peru, respectively, contained the ParC S83N mutation, and it is unclear if this mutation can cause an increased moxifloxacin MIC and treatment failure (Shipitsyna et al., 2017; Machalek et al., 2020; Manhart and Jensen, 2020). Previous studies have indicated that ParC S83R, S83I, D87N, and D87Y are most commonly associated with moxifloxacin treatment failure or *in vitro* resistance, and ParC S83I appears to show the strongest association with moxifloxacin treatment failure (Machalek et al., 2020; Manhart and Jensen, 2020; Murray et al., 2020a,b) and rapid POCTs detecting only ParC S83I might be very valuable (Sweeney et al., 2022). In contrast, detection of ParC S83N may overestimate the prevalence of resistance to moxifloxacin. There are very few options for treatment of symptomatic MG infections with combined macrolide and quinolone resistance. Pristinamycin, minocycline or doxycycline can be used as alternative treatment (Jensen et al., 2022), however, these treatment regimens do not cure all MG cases and pristinamycin is not available in many countries. This necessitates the search into new treatment strategies and/or antimicrobials. In addition, despite anorectal testing for MG in MSM enabling detection of more MG infections, the 2021 European guideline on the management of MG infections recommends testing only in men with symptomatic proctitis where other aetiologies have been excluded, because there is a high risk of dual resistance in this population (Jensen et al., 2022). It has also been shown that, based on the limited effective treatment alternatives, offering MG screening to also asymptomatic MSM might slightly decrease the MG prevalence and incidence. However, this may additionally increase the selection of AMR and result in other negative consequences related to AMR and clinical management (e.g., unnecessary psychological morbidity from infections that do not need treatment; Ong et al., 2021).

The present study evaluated MG AMR data from two African (Morocco, South Africa) and two Latin American (Guatemala, Peru) countries. Few studies from Africa have been published hitherto, all from South Africa. One study, testing samples from STI and HIV patients in 2007–2014, revealed no MRAMs, and only one (0.4%) sample with QRAM (ParC D87Y) (Muller et al., 2019). In 2011–2012, no MRAMs or QRAMs were found in HIV-positive women (Ong et al., 2019), but 9.8% of samples from sexually active women contained MRAMs (Hay et al., 2015). In 2015–2019, a single (1.1%) sample positive for both MRAMs and QRAMs was detected in men and women examined (Laumen et al., 2021). Finally, in 2015–2018 three (1.7%) samples containing MRAMs but no samples containing QRAMs were found among specimens from symptomatic primary health care centre attendees (adult women and men with genital discharge; Mahlangu et al., 2022). Our present study identified low levels of MRAMs (2.4%) and relatively low levels of QRAMs (3.7%) among women at-risk attending a youth clinic in South Africa. This is the first study to estimate MG AMR in Morocco, Guatemala and Peru. Despite wide use of 1 g single dose azithromycin in combination therapy for syndromic management of STIs in African and Latin American countries, we observed relatively low levels of MRAMs in women at-risk in South Africa, Guatemala and Morocco (2.4–11.6%). However, the prevalence of MRAMs among MSM in Peru was extremely high (65.9%). QRAM prevalence was relatively low in these countries, both in women at-risk (0–6.7%) and in MSM in Peru (5.6%). There is growing evidence that being MSM and recently having had an STI are strongly associated with MG MRAMs (Couldwell et al., 2018; McIver et al., 2019; Bradley et al., 2020; Berçot et al., 2021; De Salazar et al., 2021; De Baetselier et al., 2022), which can be attributed to the high prevalence of asymptomatic (frequently undiagnosed) MG infection, as well as frequent testing for other STIs leading to widespread use of single-dose azithromycin treatment for CT and NG. Notably, the prevalence of both MRAMs and QRAMs was substantially higher among MSM compared to women at-risk; however, these differences were also affected by the different countries where study participants were enrolled and that the level of success in the detection of MRAMs and QRAMs in the South African vaginal specimens were significantly lower (Table 2), accordingly, no straight-forward comparison between populations can be performed.

High prevalence of also the other non-viral STIs (as single STIs or in combinations) were recorded in both MSM and women at-risk. All these prevalence estimates largely exceeded the WHO population-based estimates for the corresponding regions (Rowley et al., 2019). Most of the study settings mainly use syndromic management for CT, NG and TV, which results in a lack of etiologic diagnosis and a vast overuse and misuse of antimicrobials for symptomatic STIs. Furthermore, this results also in a lack of detection and treatment of asymptomatic STIs, which effectively fuels the spread of the STIs. In these settings, it would be valuable to urgently implement more use of etiological diagnosis of non-viral STIs, such as CT, NG, TV, and MG, and increase the testing of both symptomatic and asymptomatic individuals, i.e., in line with evidence-based STI guidelines. Furthermore, strengthened preventive measures (e.g., information campaigns, promoted improved sexual behaviour and increased condom use); effective, accessible and affordable STI diagnosis and treatment; sexual contact tracing (including testing and treatment of contacts); and improved epidemiological surveillance would also be of high value.

The strengths of this study included the relatively large numbers of samples from five countries in four different WHO regions (on three continents), that mostly lack MG prevalence and AMR data. These samples were tested for the prevalence of MG, as well as coinfections with CT, NG, and TV, using the highly sensitive and specific Aptima assays (Hologic), which have been shown to have substantially higher sensitivity compared to diagnostic PCRs for STI detection (Unemo et al., 2018; Shipitsyna and Unemo, 2020; Salado-Rasmussen et al., 2022). Furthermore, this study is the first to evaluate the prevalence of MG and MG AMR in high-risk populations from four countries (Malta, Peru, Morocco, and Guatemala). The limitations included the fact that no appropriate comparison of the prevalence data for MG and AMR could be performed between MSM and women at-risk at country-level, because both populations were not targeted for testing in any single country. Other limitations were: (i) the high failure rate in the AMR testing of some of the MG-positive Aptima samples, i.e., especially in the South African samples (possibly affected by the use of the SpeeDx ResistancePlus MG assay for MRAM detection, which has a high sensitivity from Aptima MG samples (Murray et al., 2020a,b), in samples from the other countries and performance of some MRAM and QRAM testing after sample storage >3 months), and (ii) the lack of data on treatment outcome for the women at-risk and MSM. Ideally, surveillance of both MG AMR-associated mutations and treatment failures should be performed.

In conclusion, MG is prevalent internationally and enhanced aetiological MG diagnosis, linked to clinical MRAM detection, in symptomatic individuals should be implemented, where feasible (Jensen et al., 2022). Effective containment of rapidly growing MG AMR requires representative surveillance of the prevalence of MRAMs and QRAMs in MG as well as MG treatment outcome at local, national and international levels. In MSM, testing anorectal swabs in addition to urine samples enables detection of approximately 70% more MG-infected men. However, extremely high prevalence of MRAMs in MSM support avoiding screening for and treatment of MG in asymptomatic MSM (Ong et al., 2021) and in general for asymptomatic individuals (Jensen et al., 2022). Growing levels of dual resistance necessitates the development of novel therapeutic antimicrobials and therapeutic strategies, such as resistance-guided sequential therapy (Jensen et al., 2022), for MG infections. Ultimately, an effective MG vaccine might become essential.

## Data availability statement

The original contributions presented in the study are included in the article/Supplementary material, further inquiries can be directed to the corresponding author.

## Ethics statement

The studies involving human participants were reviewed and approved by the corresponding Ethics Boards at the participating countries/settings, with written or verbal inform consent obtained from all study participants. Written informed consent for participation was not required for this study in accordance with the national legislation and the institutional requirements.

## Author contributions

ES, RK, KB, IT and MU designed and initiated the study. ES and MU coordinated the study, analysed all the data and wrote the manuscript. RK, SV, VP, CA, HO, AH and EC managed the study participants and provided Aptima samples for diagnosis. DG, EM and RH were responsible for the laboratory testing. ES, RK, DG, EM, SV, RH, VP, CA, HO, AH, EC, KB, IT and MU read, commented on and approved the final manuscript. All authors contributed to the article and approved the submitted version.

## Funding

In addition to Aptima tests provided by Hologic (see conflict of interest), the study was supported by the Örebro County Council Research Committee and the Foundation for Medical Research at Örebro University Hospital, Örebro, Sweden.

## Conflict of interest

Hologic provided the Aptima *M. genitalium*, Aptima Combo 2, and Aptima *T. vaginalis* tests (special thanks to Philip Mueller and Damon Getman). However, no financial support for the testing was obtained, and Hologic had no role in data collection, analysis, interpretation or writing the paper.

## Publisher’s note

All claims expressed in this article are solely those of the authors and do not necessarily represent those of their affiliated organizations, or those of the publisher, the editors and the reviewers. Any product that may be evaluated in this article, or claim that may be made by its manufacturer, is not guaranteed or endorsed by the publisher.

## Footnotes

^1^ https://www.who.int/teams/sexual-and-reproductive-health-and-research-(srh)/areas-of-work/sexual-health/sexually-transmitted-infections/point-of-care-tests
